# Premature Burial

**Published:** 1898-02

**Authors:** 


### PREMATURE BURIAL.

There are probably few persons endowed with lively imaginations who have not at some time in their lives had grewsome fancies about being buried alive; that is, if the idea has ever been suggested to them. It forms the theme of many sensational tales of fiction, and is so much a matter of common belief that few would be disposed to question its possibility, or its occasional occurrence. In certain continental European cities this possibility is so fully credited that waiting vaults, or guarded and watched mortuaries, are kept up at public or private expense in the cemeteries, where all bodies are kept for a certain period before interment, so as to prevent any such occurrence. These are not generally so managed as to insure absolute safety from such accidents, yet they relieve the public apprehension, and may under certain circumstances be occasionally, though it must be very rarely, effective in preventing a premature burial.

In one of our English contemporaries, the *Medical Press and Circular*, there have recently appeared some articles and communications on this subject which indicate the interest the subject excites in the medical profession, as well as the difference of opinion that exists in regard to it. One writer doubts that a single well-authenticated case of actual burial of a living person has ever been satisfactorily proven; others consider it not only a possible, but a much more common, occurrence than is generally supposed. From what we know of the possibilities of the trance conditions one is hardly prepared to absolutely deny that burial might occur before death, but that this has often occurred except it may be in times of panic from epidemics is questionable enough, and Dr. Walsh is justified to a certain extent in his doubts as to the evidence in the reported cases. The fact that one writer claims to have received sixty-three letters from persons who have escaped living burial is of less weight, inasmuch as no details are given, and the natural love of narrating the marvelous or the unusual is a factor to be calculated with in such testimony.

That premature burial may occur, however, is in its way a presumption that it has occurred and may happen again under circumstances that favor such a dreadful accident. The difficulty in some cases of determining whether the condition is actual death or only a suspension of the vital activities with a possibility of their full reinstatement is very great, and, it is claimed by some, is practically impossible under ordinary conditions and with the usually available means. The possibility of premature burial can therefore be reasonably admitted, and all the more so in cases of epidemics that cause genereral panic and hurried and unceremonial interments, and also in cases of infectious diseases occurring sporadically but which for reasons of public safety require a hasty burial. The only real, universally practical test is commencing decomposition, though others are generally possible and effective, and it is therefore impossible that such an accident as the burial of a living person can often occur. The experience of the waiting or observation mortuaries in some European cities supports this view, though their continuance implies that the belief in their utility maintains itself in spite of the rarity of occasions.

The most elaborate provision against living burial is one recently announced by a Mr. Carnis of Paris, which he calls "Karnice," and which is to render all burials provisional, as it were, for the person entombed, while still inviolate to all the rest of the world. The apparatus is to be attached to the coffin for a certain period after interment, is set in action by the least movement of the supposed corpse; while impossible to be tampered with by outside parties it gives an alarm and admits light and air to the buried person; is cheap and easily adapted, and can be used repeatedly, so that its employment would only cost a slight rental, and a few such apparatuses would suffice for a large cemetery. If it is all that its inventor claims, it would appear to be almost an absolute safeguard against death from premature interment. On the other hand, it would seem that it might be liable to give false alarms, if by gaseous decomposition or any other cause a movement of the corpse should take place, though this might perhaps not outweigh or seriously affect its value in the minds of those fearing the accident of being buried alive. It is not improbable that many of the dislocations or derangements of bodies, found on exhumation, are due to the causes alluded to and the possibility of their occurrence should always be taken into account.

The horror of finding one's self in a living tomb appeals directly to the imagination of every one; it seems the most terrible of all possible deaths. There is, however, another way to look at it when the physical conditions are considered. The air space in a tight coffin under five feet of earth is limited, not over two or three cubic feet at the utmost, outside of the space ordinarily occupied by the body. Unless consciousness returned simultaneously with respiration, asphyxia might possibly precede it and annul it—there might be some evidences of convulsion that might be interpreted as voluntary movement, but they are not necessarily indicative of such. The cases where consciousness exists throughout must be rare, though they have been made much of in fiction, and are popularly believed to be almost the rule. It is no comfort to think that any one can be buried alive, but it is better to believe, as the facts warrant us, that the long lingering death in hopeless horror, and the powerless anticipations of the fate, are still less probable events.

With the modern methods of preservation of remains which are so often employed, living burial is impossible and we do not hear of any accidental homicides brought about in this way, and with reasonable delay and care the possibilities of premature interment are reduced in all cases to almost nothing, if not absolutely destroyed.—*The Journal.*

